# Supplementary material for: Directing curli polymerization with DNA origami nucleators
Source: Nat Commun. 2019 Mar 27;10:1395. doi: 10.1038/s41467-019-09369-6 (PMC6437208; doi:10.1038/s41467-019-09369-6)
Supplement: Supplementary file 1 — Supplementary Information [file 41467_2019_9369_MOESM1_ESM.pdf]

Supplementary Information

Directing Curli Polymerization with DNA Origami Nucleators

Mao et al

Supplementary Figures

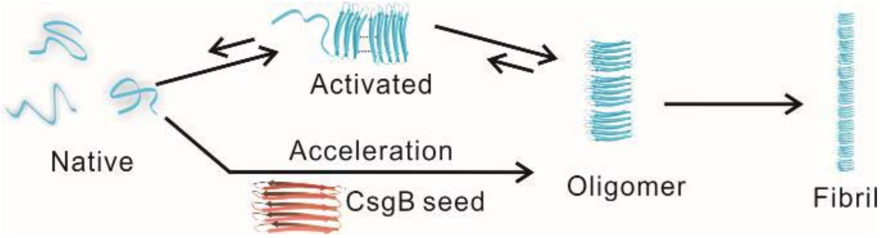

Supplementary Figure 1 | Typical nucleation pathways for CsgA fibril. In the absence of CsgB seed, CsgA proteins in solution can first fold and then form oligomer structures arising from molecular interactions among folded monomers, followed by the formation of fibrils. The subsequent aggregation of fibrils eventually leads to the formation of mature fibers. The presence of CsgB would possibly promote the oligomer formation due to more favorable molecular interactions between CsgA and CsgB.

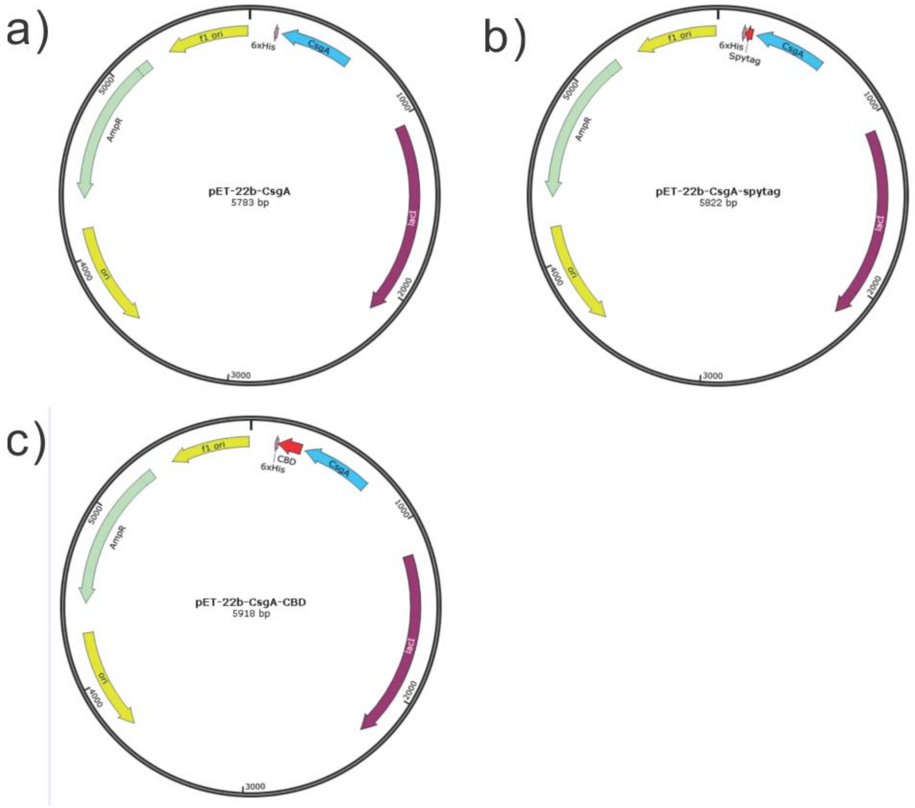

Supplementary Figure 2 | Plasmid maps of gene constructs for CsgA proteins. (a) PET22b containing CsgA; **(b)** PET22b containing CsgA-spytag; **(c)** PET22b containing CsgA-CBD.

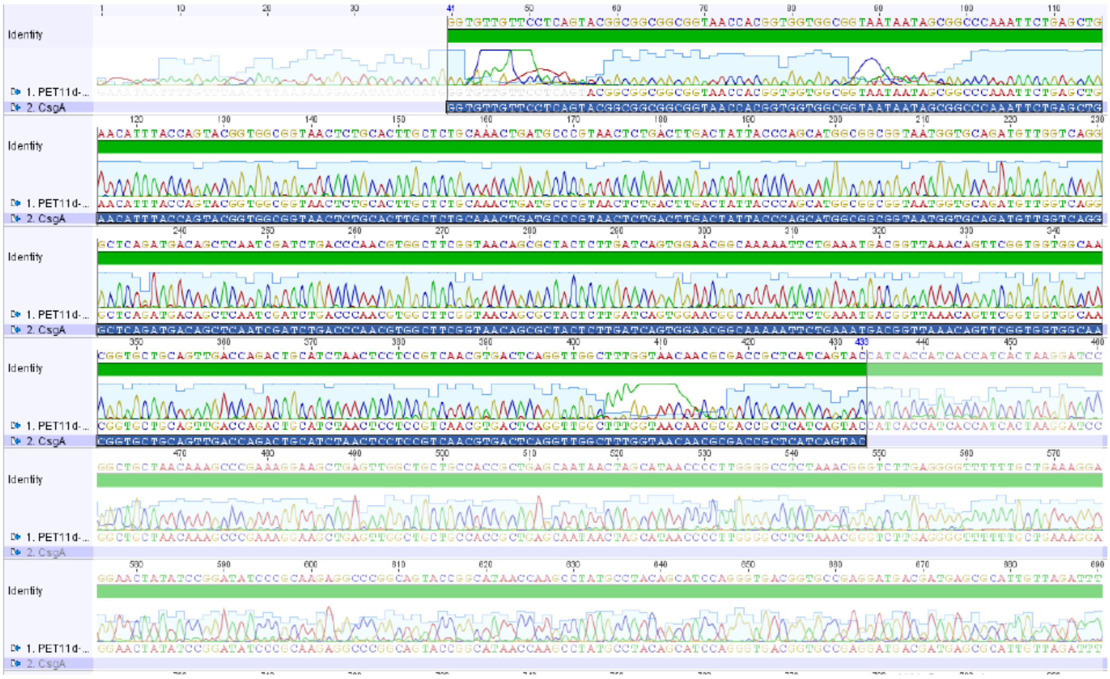

Supplementary Figure 3 | Sequencing results of the vector expressing CsgA. The consensus sequence is the sequence above the reference sequence (green line).

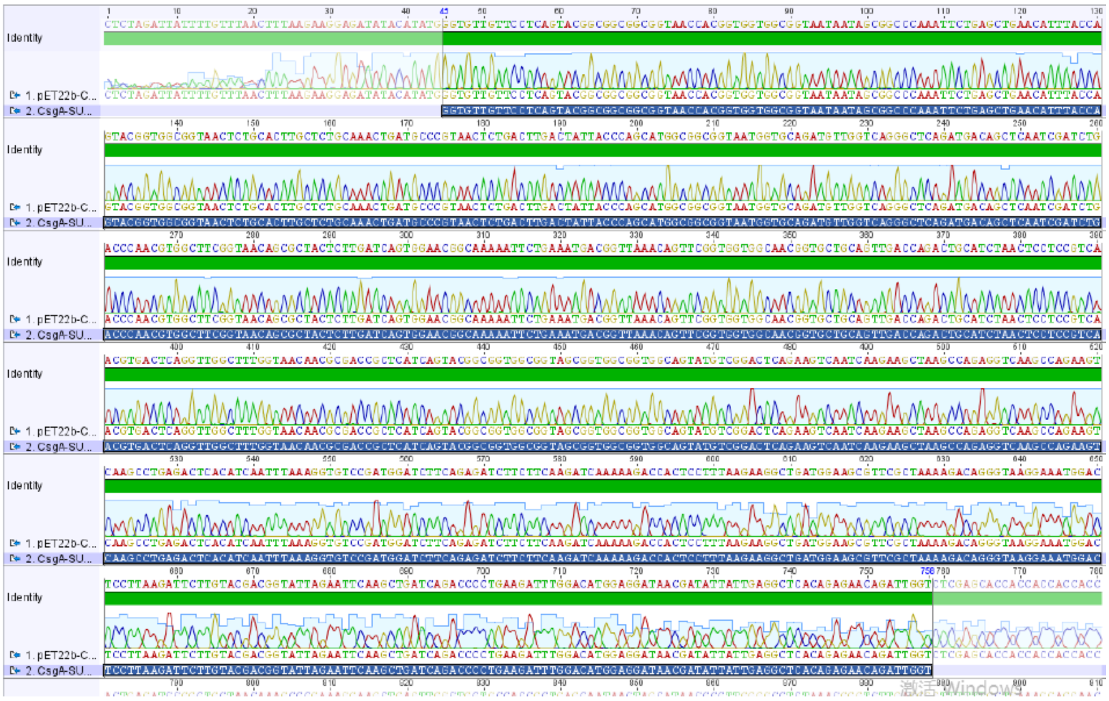

Supplementary Figure 4 | Sequencing results of the vector expressing CsgA-spytag. The consensus sequence is the sequence above the reference sequence (green line).

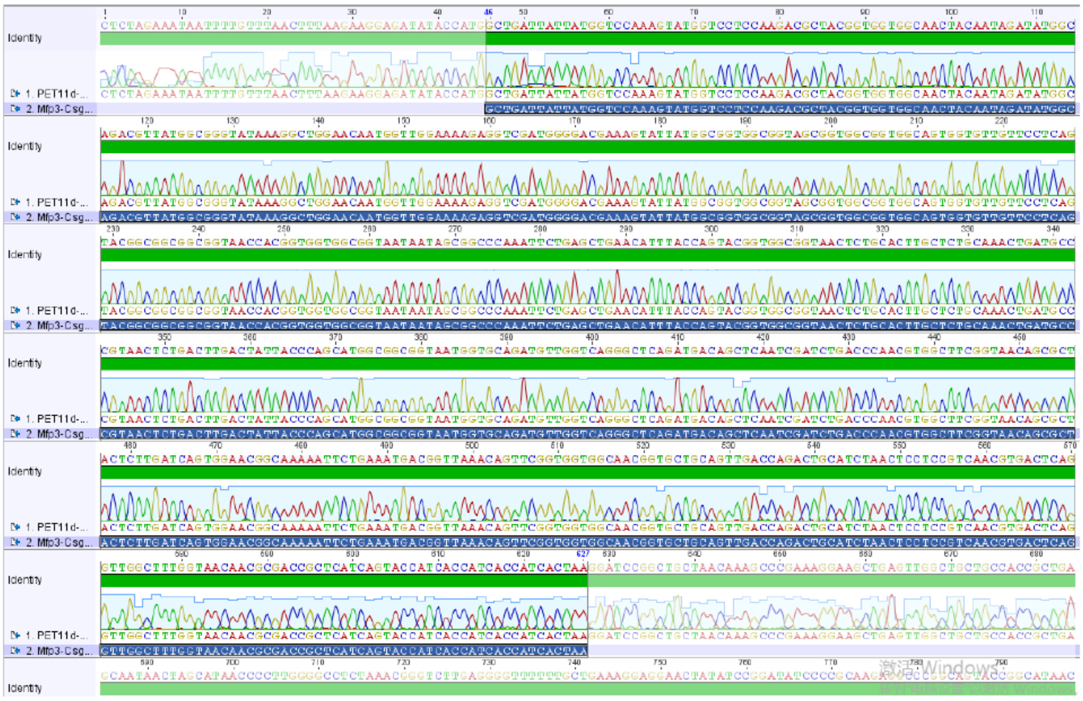

Supplementary Figure 5 | Sequencing results of the vector expressing CsgA-CBD. The consensus sequence is the sequence above the reference sequence (green line).

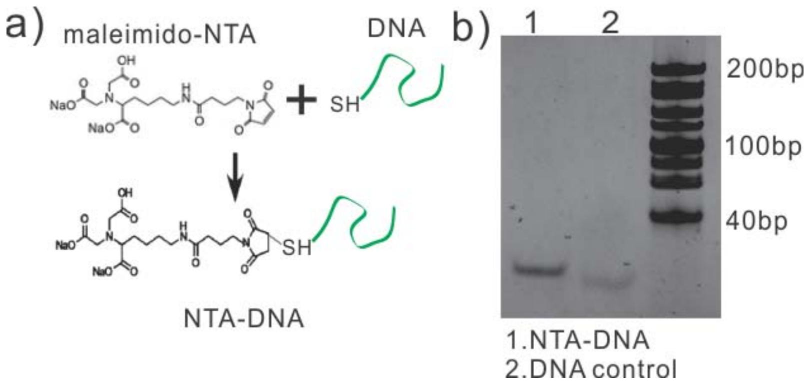

Supplementary Figure 6 | NTA-DNA conjugation. (a) Schematic showing the synthesis of NTA-DNA by chemical modification of a DNA strand containing a thiol group with maleimido-C3-NTA; **(b)** native PAGE showing the NTA modified DNA (lane 1) and DNA control (lane 2). The PAGE gel was stained with EB (Ethidium bromide). Source data are provided as a source data file.

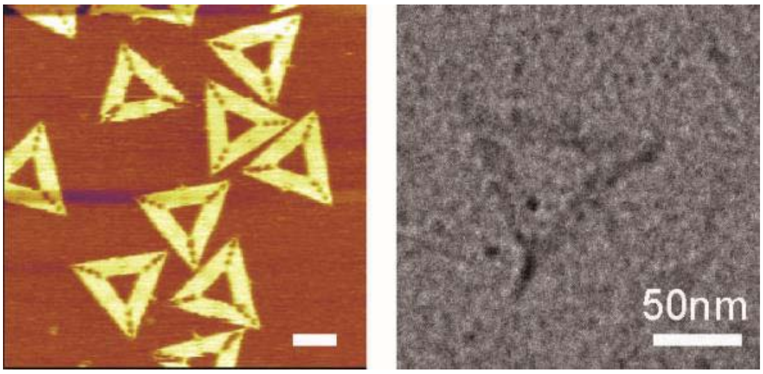

Supplementary Figure 7 | AFM and TEM image of typical triangular DNA origami. Scale bars: 50 nm.

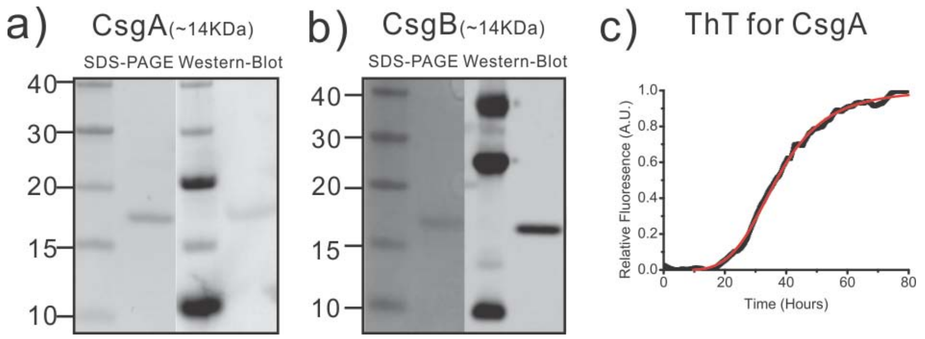

Supplementary Figure 8 | Biological assays of purified CsgA and CsgB proteins. (a) & (b) Coomassie-stained SDS-PAGE and western blots with anti-His antibodies confirm the expressed proteins: **(a)** CsgA and **(b)** CsgB (see Supplementary Note 1). Source data are provided as a source data file; **(c)** ThT assay revealing the kinetics of amyloid formation for CsgA. Source data are provided as a source data file.

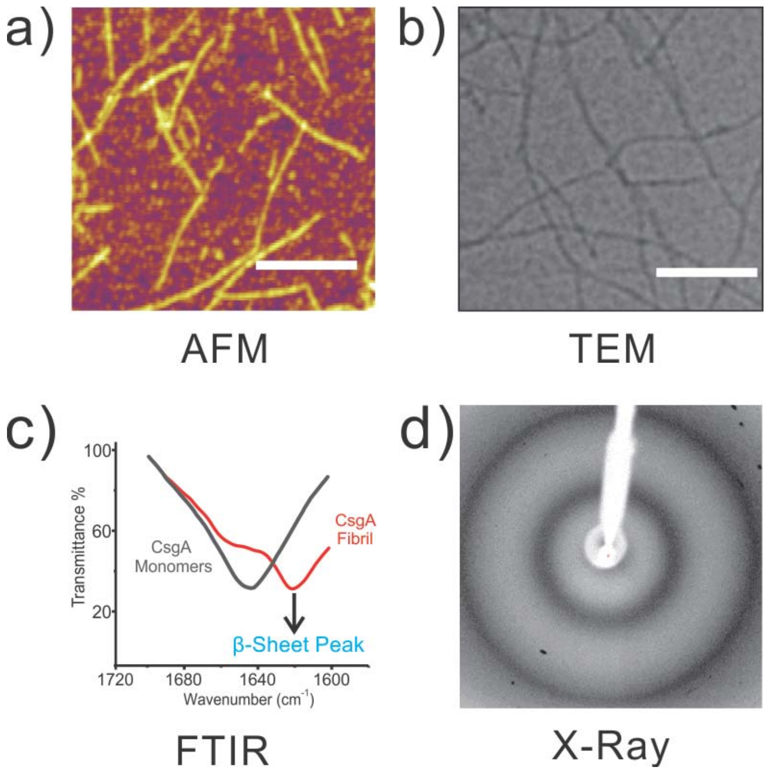

Supplementary Figure 9 | Morphological and structural characterization of CsgA fibrils. (a) Representative AFM image of CsgA fibrils; **(b)** TEM image of CsgA fibrils; **(c)** FTIR spectrum of CsgA fibrils; **(d)** X-ray fiber diffraction pattern of CsgA fibrils, a cross-β core structure with an axial reflection at 4.8 Å and an equatorial reflection at 9.0 Å arising from the inter-strand and β-sheet spacing. Samples used for characterization were CsgA fibril samples formed after incubation of CsgA solution for over 48 hours (see Supplementary Note 1). Scale bar: 100 nm.

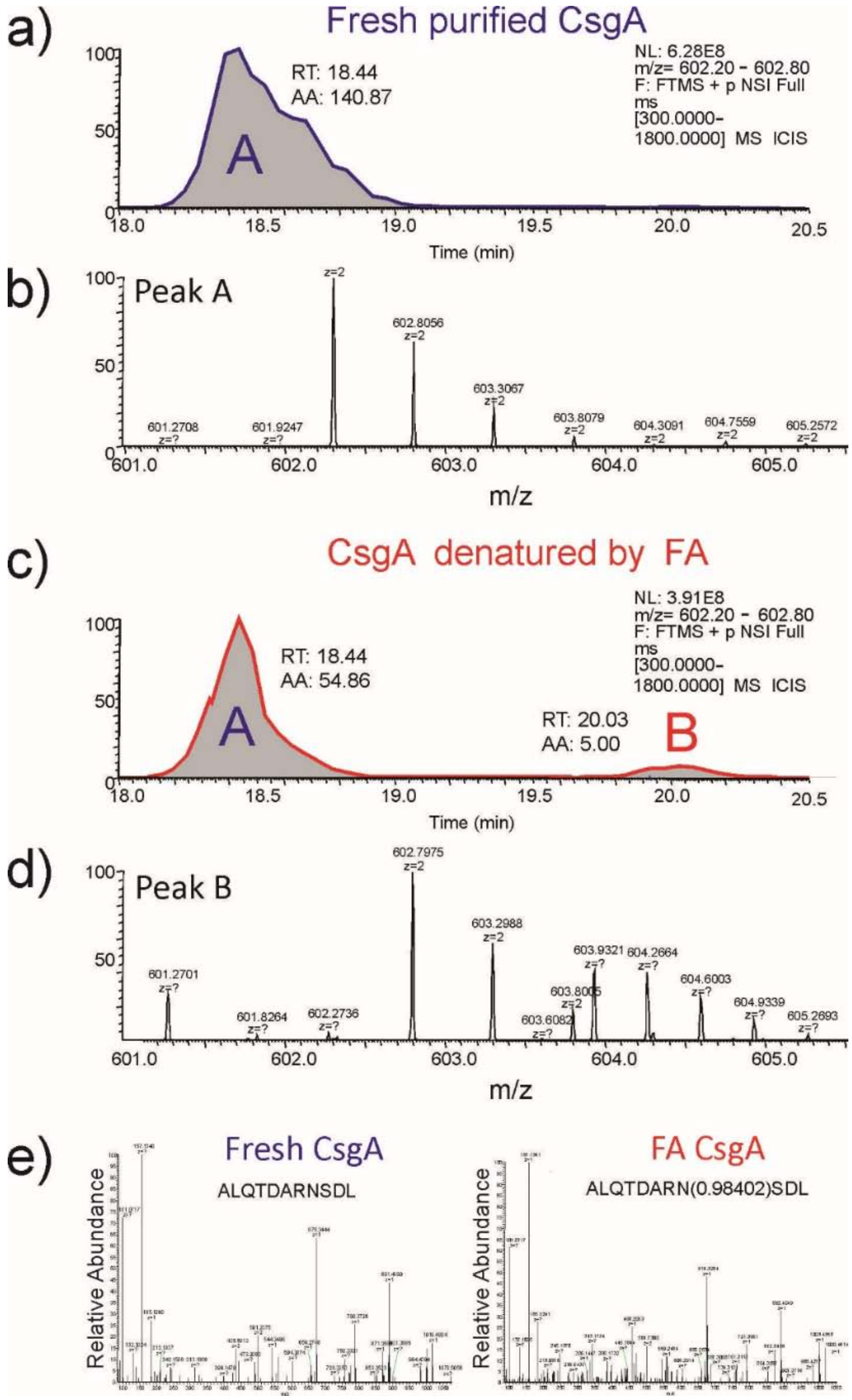

Supplementary Figure 10 | Integrity determination for CsgA with HPLC-MS. Nonenzymatic deamidation of Asn (N) is widely observed in CsgA. To evaluate the integrity of CsgA, we applied MS to determine whether this deamidation occurred during sample preparation. **(a)** & **(c)** HPLC traces for the peptide 38–48 of freshly purified CsgA and FA-stored CsgA, respectively. **(b)**, **(d)** & **(e)** Mass spectra for the peptide 38–48 of the freshly purified CsgA and FA-stored CsgA, respectively. These results suggest that most CsgA stored in Formic acid remain integrity as fresh purified CsgA, indicating that deamination process has little influence on the integrity of CsgA.

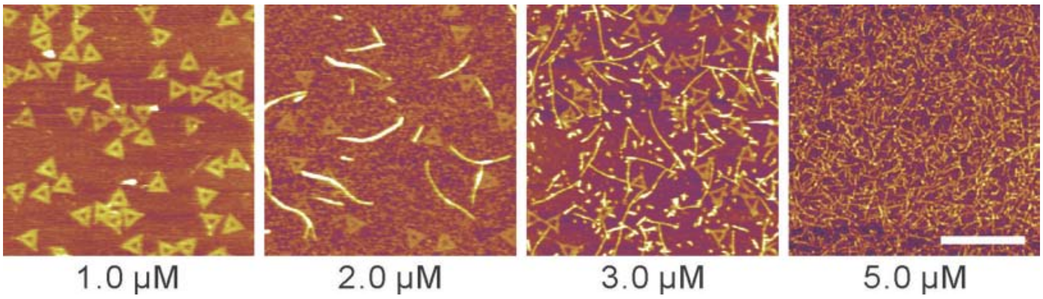

Supplementary Figure 11 | CsgA polymerization at varied concentration with CB-origami. AFM images showing the structures obtained by nucleation-directed polymerization of CsgA solutions of different concentrations in the presence of CB-origami (0.5 nM). The concentrations of CsgA applied (from left to right) were 1.0, 2.0, 3.0 and 5.0 μM, respectively (see Supplementary Note 2). Scale bars: 1 μm.

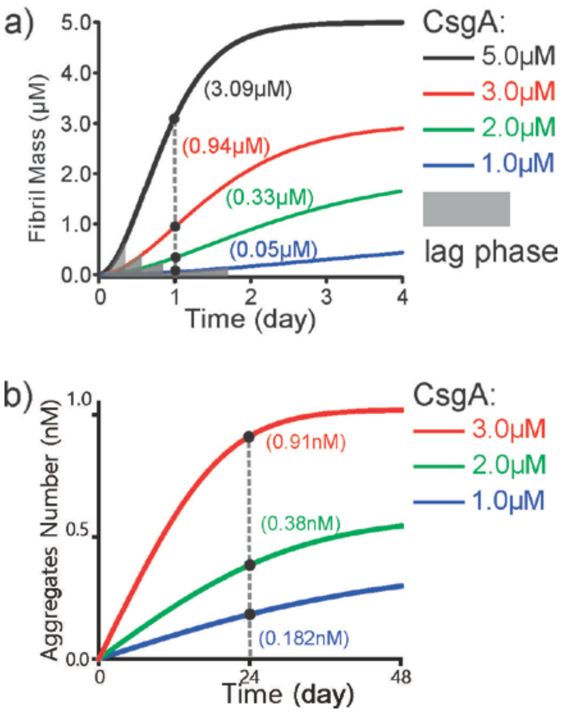

Supplementary Figure 12 | Kinetic models for CsgA polymerization with CB-origami. This model is based on the Supplementary Methods with K_{+}=5.24*10^{7} M^-1 h^-1, K_{2}= 6.49*10^{5} M^-2 h^-1, and K_{n}=2.08 M^-1 h^-1 (see Supplementary Note 3). **(a)** a kinetic mode for fibril mass vs polymerization time to explain the sharp concentration dependence in fiber extension. In this model, we calculated the produced mass of CsgA aggregates after incubating for 1 day, and found the produced mass is 3.09 μM in the 5.0 μM CsgA system (black curve), which is about 60-fold higher than that (0.05 μM) of the 1.0 μM CsgA system (blue curve). Specifically, at higher concentration, (for example, 5 μM), the polymerization already passed the initial nucleation stage and more independent fibrils formed and covered the surface of the substrate. **(b)** a kinetic model for aggregate numbers produced vs time to explain the fact that more independent fibril polymerization formed when CsgA concentration is above 3.0 μM (see Supplementary Note 4).

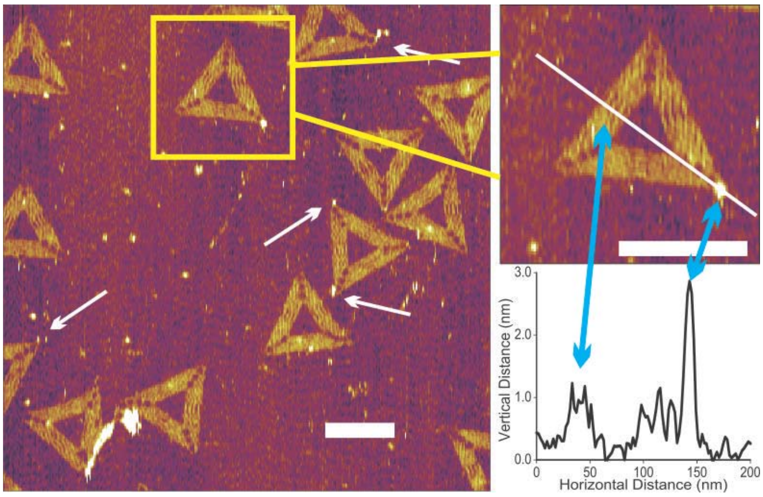

Supplementary Figure 13 | AFM morphology of early-stage aggregates with CB-origami. AFM images showing the structures obtained by nucleation-directed polymerization of CsgA solutions (1.0 μM) in the presence of CB-origami (0.5 nM). Scale bars: 100 nm.

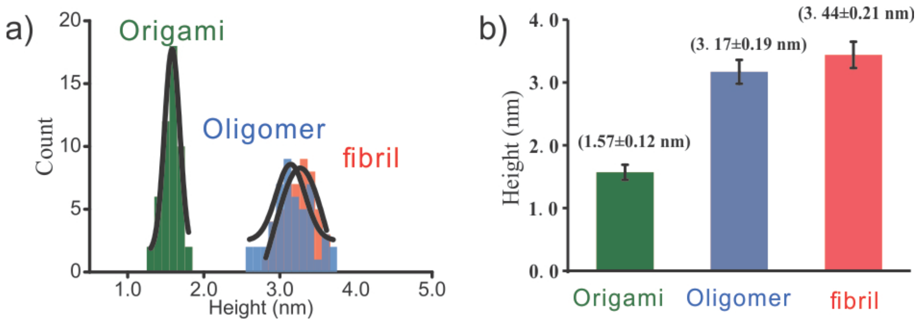

Supplementary Figure 14 | Height comparison of origami and CsgA aggregates. (a) Histograms showing the height distribution of Origami, CsgA oligomer and CsgA fibril. (**b)** Height comparison of origami., CsgA oligomer and CsgA fibril. Data were collected based on 50 counts of fibrils for each group and presented as the mean ± sd. Source data are provided as a source data file.

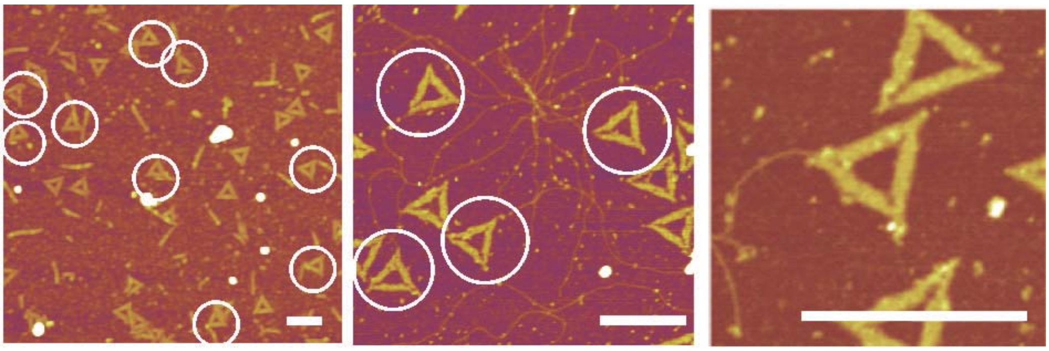

Supplementary Figure 15 | AFM characterization of CB-origami/fibril structures. AFM images showing that CsgA fibrils tethered to the origami landmark. Scale bars: 200 nm.

Supplementary Figure 16 | TEM characterization of CB-origami/fibril structures. (a)-(d) TEM images showing that CsgA fibrils were tethered to the origami landmark. Scale bars: 100 nm.

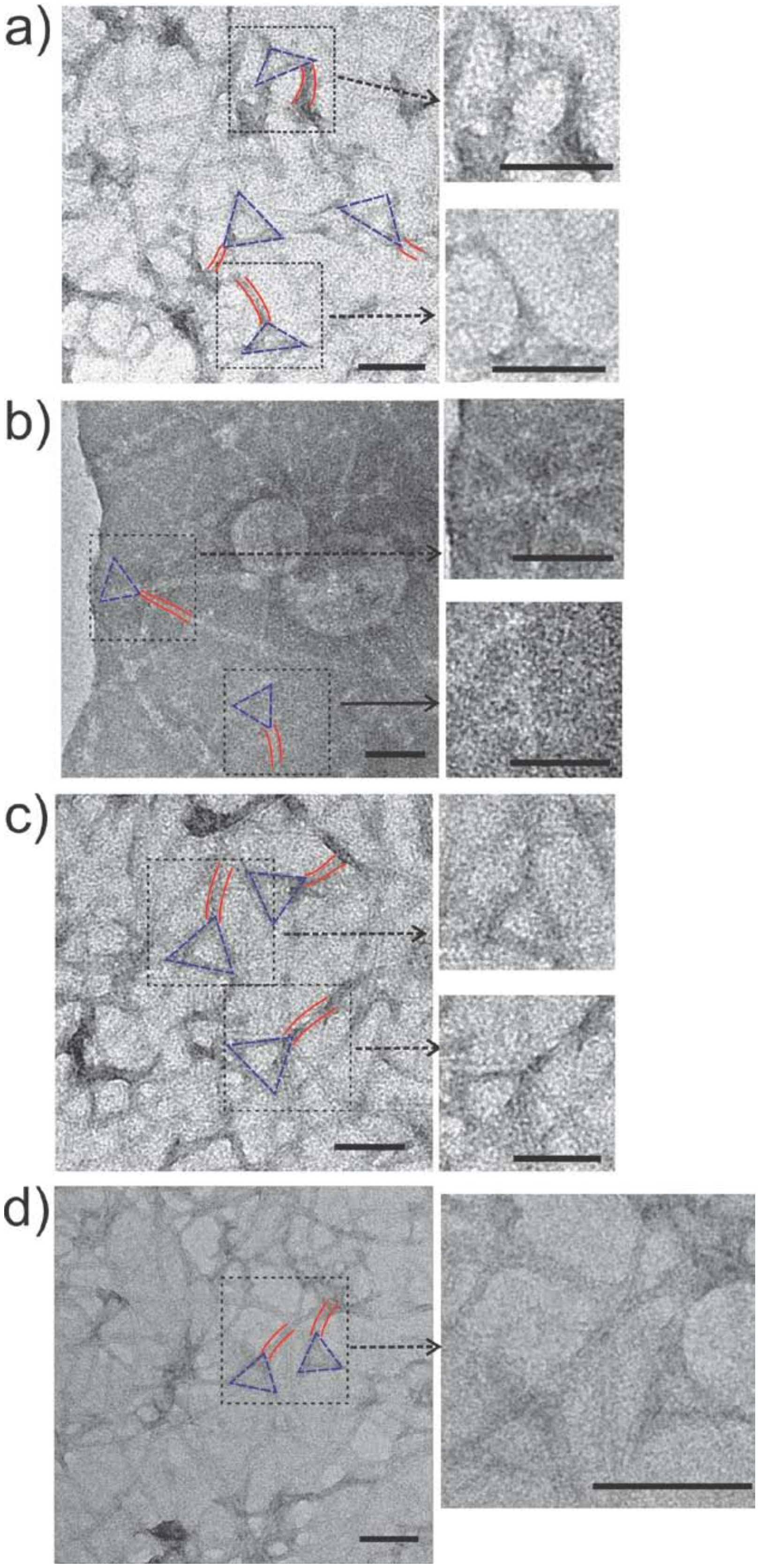

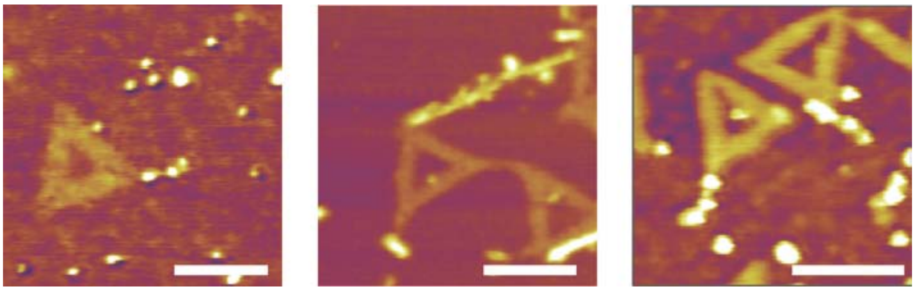

Supplementary Figure 17 | AFM morphology of CB-origami/fibrils labeled with Au NPs. AFM images showing that gold nanoparticles specifically bound to CsgA fibrils tethered to the origami landmark. Scale bars: 100 nm.

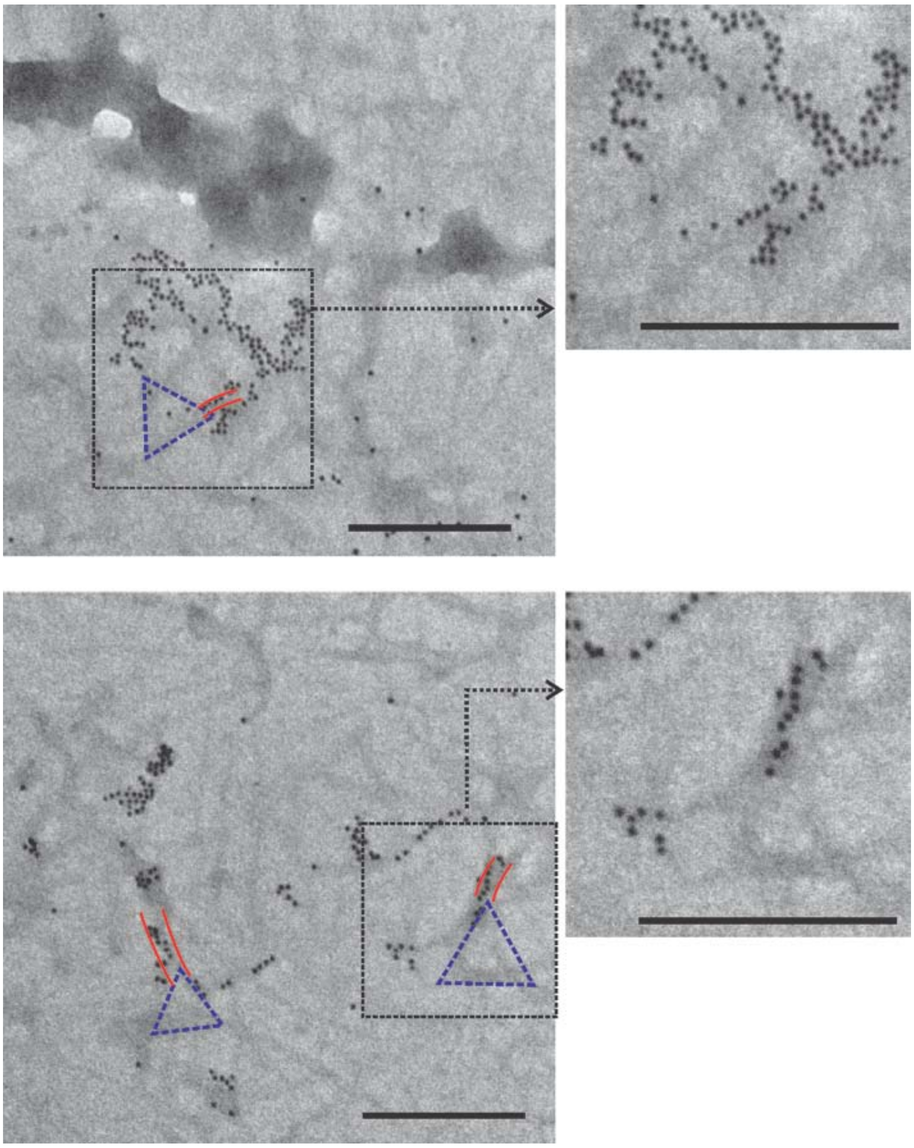

Supplementary Figure 18 | TEM images of CB-origami/fibril structures labeled with Au NPs. TEM images showing that gold nanoparticles specifically bound to CsgA fibrils tethered to the origami landmark. Scale bars: 200 nm.

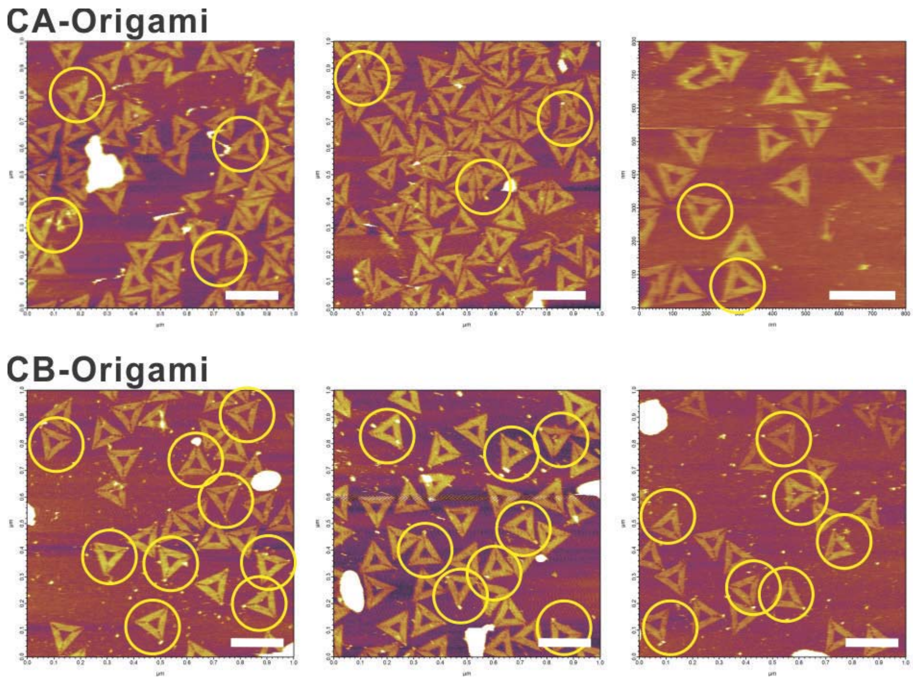

Supplementary Figure 19 | Nucleation efficiency determination for CB-origami. The nucleation efficiency for CB-origami and CA-origami (with 1.0 μM CsgA monomers). Typical AFM images for CA-origami and CB-origami incubated with 1.0 μM CsgA. The yellow circular indicates the site of formed oligomer at the vertex of origami. Scale bars: 200 nm.

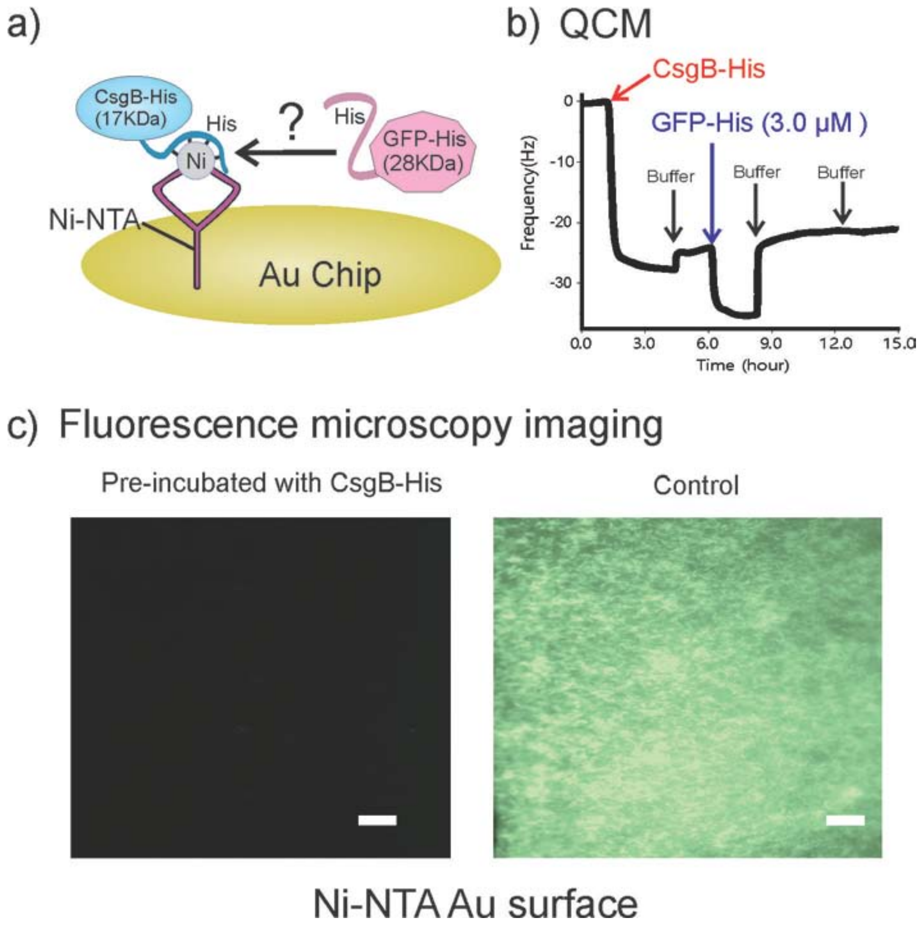

Supplementary Figure 20 | Assessing the replacement reaction between CsgA and CsgB. (a) Cartoon showing the Ni-NTA decorated Au sensor chip based assay to probe the replacement of CsgB-His (originally tethered to the Ni-NTA sites) with GFP-His (3.0 μM) based on QCM technique. **(b)** Real-time frequency response of the Au chip sensor upon exposure to different protein solutions or washing buffer under constant flow rate (25 μL/min), showing different adsorption (binding) and de-adsorption (de-binding) events. The arrows refer to the time points at which CsgB-His protein solution, (washing) buffer or GFP-His solution was flowed in (see Supplementary Note 5). **(c)** Fluorescent imaging used to determine if GFP-His could replace the CsgB-His initially tethered to the Ni-NTA decorated Au Chip. The left image aquired from half of the chip that was pre-incubated with CsgB-His solution, and the right image aquired from the other half of the chip (the control Ni-NTA decorated Au chip). The whole chip was subjected to incubation in 3.0 μM GFP-His solution before imaging. scale bars: 1 μm.

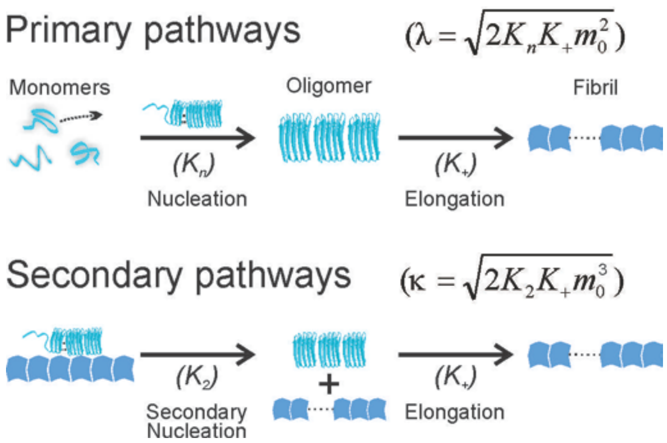

Supplementary Figure 21 | Typical polymerization pathways for CsgA. The corresponding proliferation rates representative of the primary and secondary pathways. In primary pathways, such as homogeneous nucleation, new aggregates form at a rate dependent on the concentration of monomers and independent of the concentration of existing fibrils. A combined parameter λ = (K_n K_+ m_0^2) ^(1/2) can be used to describe the proliferation rate of primary pathways. Secondary pathways are typically featured by new aggregates forming at a rate dependent on the concentration of existing fibrils. In secondary pathways, such as secondary nucleation, the surfaces of existing fibrils catalyze the nucleation of new aggregates from the monomeric state, with a rate dependent on the concentration of monomers and that of existing fibrils. The proliferation rate of secondary pathways can be described by κ = (K_2 K_+ m_0^2) ^ (1/2). Note: K_n, K_+ and K_2 stands for the kinetic rate constant for the primary nucleation, fibril elongation and secondary nucleation processes respectively, m_0 refers to initial concentration of soluble monomers.

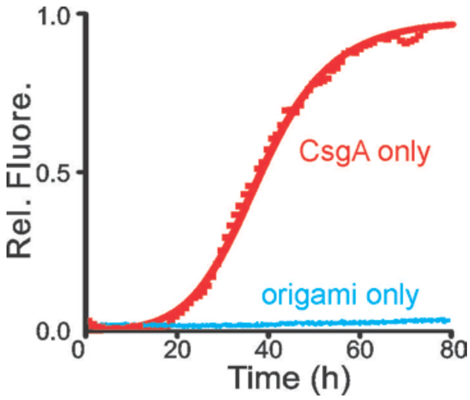

Supplementary Figure 22 | ThT Kinetics of CsgA and DNA origami alone. ThT analysis for CsgA (5 μM, red curve) and origami (10 nM, blue curve), respectively. The data (dotted curves), representative of the cumulative normalized fluorescence signal of the samples, were fitted with the equation described in the Supplementary Methods (solid-line curves)**.**

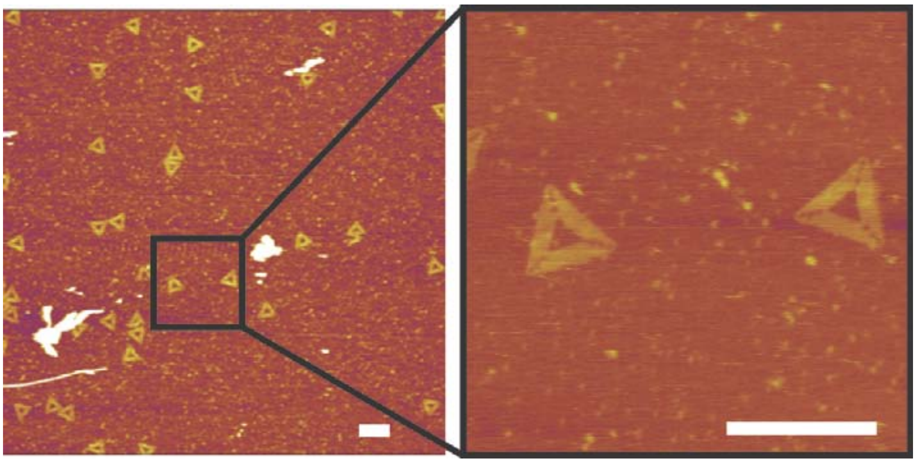

Supplementary Figure 23 | AFM images of DNA origami after ThT incubation. The AFM images showed that DNA origami remained intact for the duration of the ThT assay (with 450 nm light exposure). Scale bars: 200 nm.

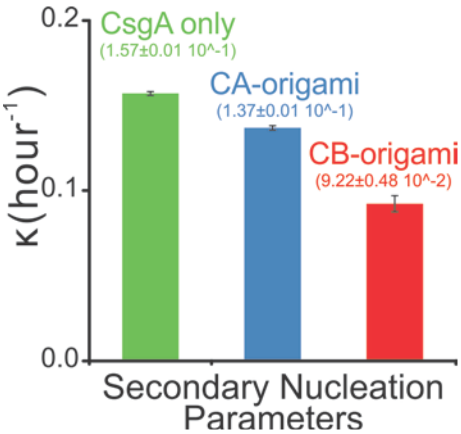

Supplementary Figure 24 | Kinetic rate comparison of secondary pathways. κ stands for the proliferation rate of secondary pathways and presented as the mean ± sd based on three independent experiments (n=3). Source data are provided as a source data file.

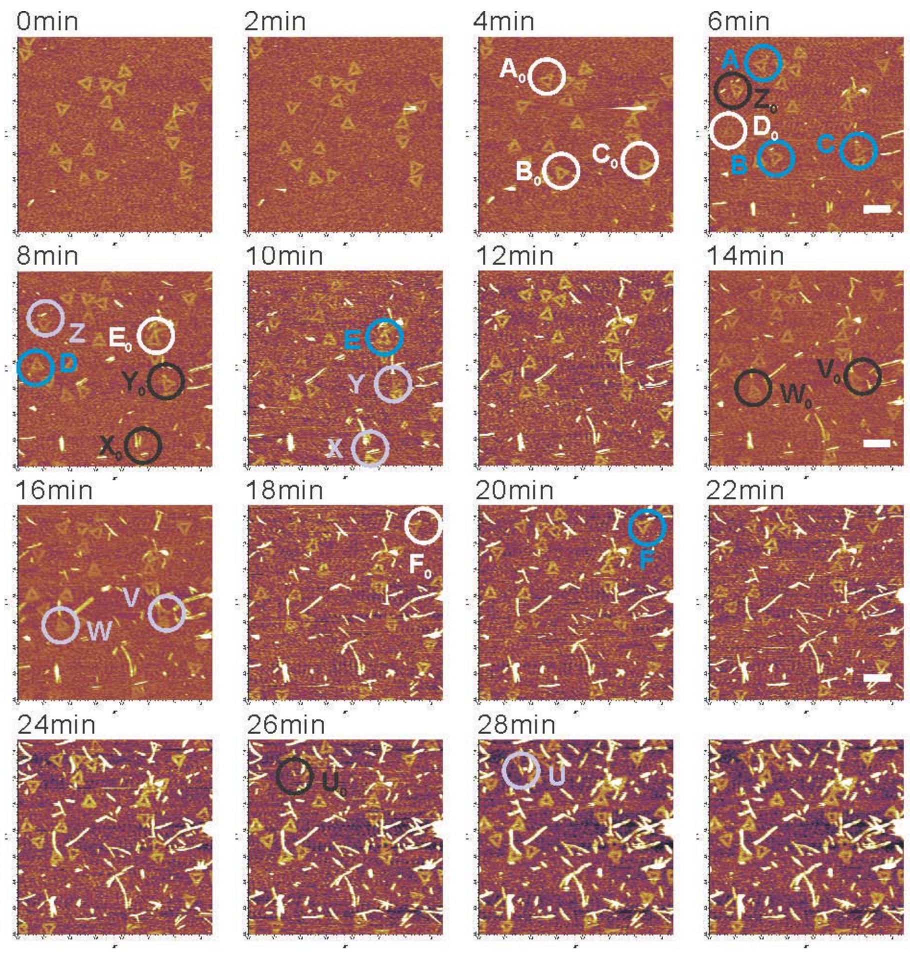

Supplementary Figure 25 | Time-lapse imaging of fibril growth at the vertices of CB-origami. The examples (A, B, C, D, E and F) for departure mode for CsgB-mediated CsgA polymerization are marked in blue and white, while the examples (Z, Y, X, W, V and U) for arrival mode are marked in black and light purple. In particular, the white and black markers indicate the initial states of departure and arrival mode, respectively. The subscript number 0 indicates the original state before in situ CsgA polymerization. Scale bars: 200 nm.

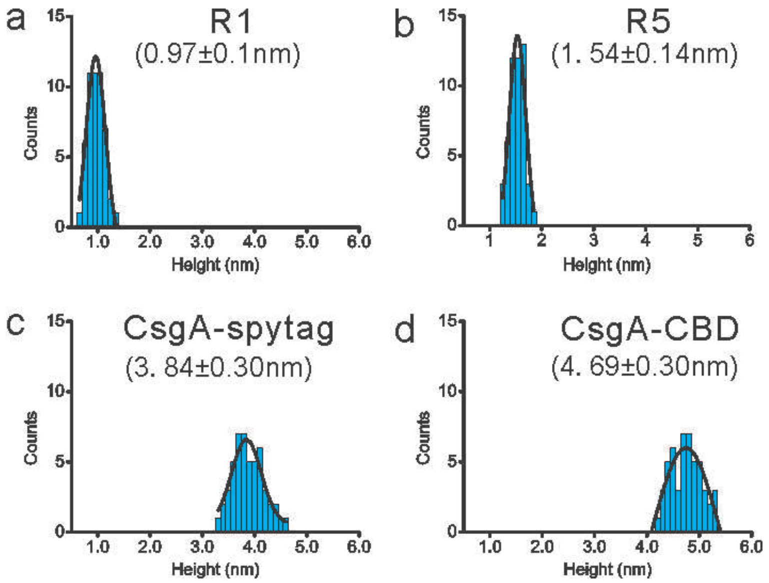

Supplementary Figure 26 | Height distribution of different types of fibrils. Histograms showing the height distribution of **(a)** R1, **(b)** R5, **(c)** CsgA-spytag and **(d)** CsgA-CBD. Data were collected based on 50 counts of fibrils for each group and presented as the mean ± s.e.m.

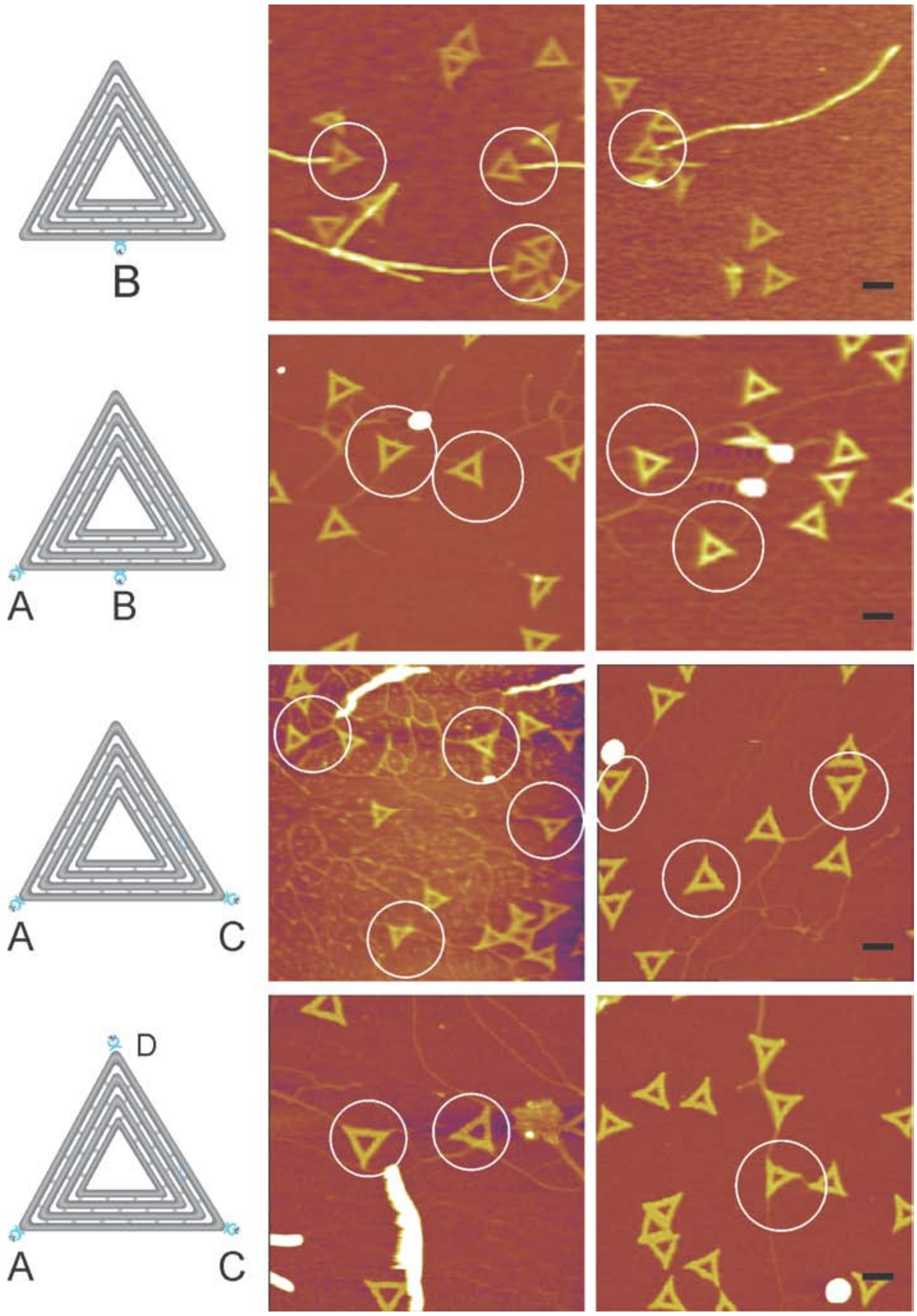

Supplementary Figure 27 | Diverse DNA origami/amyloid complex structures. AFM images of typical DNA origami-fibril complex structures formed by polymerization of CsgA monomers (2.0 μM) in the presence of designed CB-origami, with CsgB tethered at one, two or all of the three vertexes (see Supplementary Note 6). Scale bars: 100 nm.

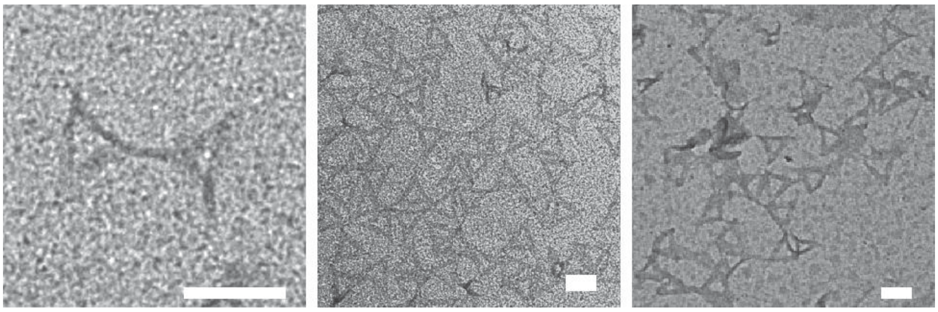

Supplementary Figure 28 | TEM images of DNA origami/fibril complex structures. DNA origami-fibril complex structures formed by polymerization of CsgA monomers (2.0 μM) in the presence of CB-origami, with CsgB tethered at the three vertexes. Scale bars: 100 nm.

Supplementary Tables

Supplementary Table 1. Comparison of elongated distance for different elongation mode. Source data are provided as a source data file.

Mode	Peak position(nm)	FWHM(nm)
Departure	4.7±2.0	15.2
Arrival	5.3±0.6	10.9
Stochastic	6.4±0.4	7.7

Supplementary Table 2. Comparison of stagnation time for different elongation mode. Source data are provided as a source data file.

Mode	Peak position (min)	FWHM (min)
Departure	0.26±0.07	0.34
Arrival	0.22±0.03	0.32
Stochastic-a	0.59±0.04	0.66
Stochastic-b	2.07±0.06	0.84

Supplementary Table 3. Amino-acid sequences used in this study.

CsgA-HisTag	GVVPQYGGGGNHGGGGNNSGPNSELNIYQYGGGNSALALQTDARNSDLTITQHGGGNGADVGQGSDDSSIDLTQRGFGNSATLDQWNGKNSEMTVKQFGGGNGAAVDQTASNSSVNVTQVGFGNNATAHQYHHHHH
CsgA-Spytag-HisTag	GVVPQYGGGGNHGGGGNNSGPNSELNIYQYGGGNSALALQTDARNSDLTITQHGGGNGADVGQGSDDSSIDLTQRGFGNSATLDQWNGKNSEMTVKQFGGGNGAAVDQTASNSSVNVTQVGFGNNATAHQYGGGGSGGGGSAHIVMVDAYKPTKHHHHHH
CsgA-CBD-HisTag	GVVPQYGGGGNHGGGGNNSGPNSELNIYQYGGGNSALALQTDARNSDLTITQHGGGNGADVGQGSDDSSIDLTQRGFGNSATLDQWNGKNSEMTVKQFGGGNGAAVDQTASNSSVNVTQVGFGNNATAHQYGGGGSGGGGSAWQVNTAYTAGQLVTYNGKTYKCLQPHTSLAGWEPSNVPALWQLQHHHHHH
R1 domain from CsgA	SELNIYQYGGGNSALALQTDARN
R5 domain from CsgA	SSVNVTQVGFGNNATAHQY

Supplementary Methods

Plasmid construction

The pET-22b vector was cleaved at XhoI and NdeI sites. The genes for CgA, CBD and spytag were separately amplified by PCR with introduced compatible overhangs for Gibson assembly. The cleaved vector and appropriate PCR products were mixed and incubated with Gibson Assembly Master Mix at 50°C for 1 h, then transformed into DH5a *E. coli* cells. PCRs were carried out with primer oligos from by Genewiz Inc. A Bio-Rad S1000 Thermal Cycler with Dual 48/48 Fast Reaction Modules (Bio-Rad) was used to perform PCRs, ligation and Gibson Assembly. Gel extractions were carried out with Gel Extraction Kits (Tiangen Inc). All constructs were sequence verified by Genewiz, and the sequencing results for all genes are presented in Supplementary Figure 2 - 5.

Protein expression and purification

The recombinant plasmids were transformed in BL21(DE3). A single colony was inoculated into 5 mL of LB broth containing 50 mg/mL ampicillin and incubated overnight in a shaker at 37 °C. The overnight culture was inoculated into 5 L broth containing 50 mg/mL ampicillin and grown at 37 °C with vigorous shaking until the OD600 reached 0.5-0.7. The shaker was then adjusted to the designated expression temperature. Protein expression was induced with 0.5 mM IPTG at 37°C for 1 h. Cells were collected by centrifugation. 5 gram cell pellets were resuspended and lysed by 50 mL extraction solution (8 M guanidine hydrochloride (GdnHCl), 300 mM NaCl, 50 mM K_2HPO_4/KH_2PO_4, pH 7.2). Lysates were incubated at 4°C for 24 h. The insoluble portions of the lysates were removed by centrifuging at 10,000 g, and the supernatants were incubated with 6 mL Ni-NTA (thermal) column for 2 h at room temperature. Beads that bound His-tagged CsgA and its derivatives were then centrifuged, and washed with 20 mL of potassium phosphate buffer (300 mM NaCl, 50 mM K_2HPO_4/KH_2PO_4, pH 7.2) for two times. The washed beads were then added with 3 mL potassium phosphate buffer (300mM NaCl, 50 mM K_2HPO_4/KH_2PO_4, pH 7.2). The mixed solutions were then loaded on the columns (Sangon 2-mL disposable gravity column). GdnHCl was further washed away by adding another 12 mL potassium phosphate buffer (300mM NaCl, 50 mM K_2HPO_4/KH_2PO_4, pH 7.2). 6 mL washing buffers (40 mM imidazole and 50 mM potassium phosphate buffer, pH 7.2) were passed through the columns to remove contaminated proteins with 5 consecutive washing steps[1].

The collected elution buffer containing the purified monomer proteins was incubated for over 3 days to allow self-assembly of CsgA proteins into fibrils. The fibril precipitate was collected, resuspended in 98% formic acid, and air-dried with N_2 gas. The N_2 gas dried with powder was then suspended in 1 × HEPES-Mg^{2+} solution (10 mM HEPEs and 12.5 mM Magnesium acetate, pH 8.0). The solution was then filtered with a 220 nm CA SYR FILTER (Thermo Scientific) to remove the undissolved aggregate. The remaining solution containing protein monomers was then used for probing nucleation-directed polymerization in this study.

Conjugation of NTA-DNA

The DNA strands with protected thiol groups (“Thio”) on their 3’ or 5’ ends used in this experiment were purchased from Takara Inc (HPLC grade). DTT (DL-Dithiothreitol) treatment was performed immediately before conjugation to remove the C3 protection group to generate a thiol moiety. Maleimido-C3-NTA was purchased from Dojindo Molecular Technologies. 1mM

Maleimido-C3-NTA was then added to 10 μM DNA solution in 10 mM HEPES (pH 7.3, thermal) and 50 mM NaCl (thermal) buffer solution (0.5 mL). The reaction was maintained overnight at room temperature. After the reaction, the excess of maleimido-C3-NTA was then removed from the solution by Microcon centrifugal filtration devices (3 kD MWCO filters, Millipore, Bedford, MA).

Design and preparation of DNA origami structures

To design DNA origami with multiple anchoring sites of CsgB at specific positions, we replaced certain staple DNA strands in the DNA origami with DNA capturing strands in a site-defined manner.

To assemble the triangular DNA origami, 5 nM single stranded M13mp18 DNA (NEB, 7,249 nt long) was mixed in 1×HEPES-Mg^{2+} (10 mM HEPEs and 12.5 mM Magnesium acetate, pH 8.0) with staple strands (from Jieli Inc) and capture strands in a 1 : 10 : 20 molar ratio, following the design originally proposed by Rothemund[2]. The resulting solution was cooled from 95°C to 4°C at 0.2^{o}C/min to form the NTA-origami.

Formation of CB-origami

The NTA-origami (5 nM) decorated with NTA-DNA strand was incubated with 1.0 μM CsgB-His proteins and 5 μM $NiCl_2$ solution for over 1h to form CB-origami. The resultant solution was subsequently purified with a Microcon centrifugal filtration device (100 kD MWCO filters, Millipore, Bedford, MA) to remove the excessive CsgB and Ni^{2+}. A similar procedure was applied to produce CA-origami.

CB-origami directed polymerization of CsgA proteins

5 μL diluted CB-origami (with 1 nM CsgB monomer concentration) in 1×HEPES-Mg^{2+} (10 mM HEPEs and 12.5 mM Magnesium acetate, pH = 8.0) was deposited on freshly cleaved mica (SPI Supplies, 9.5 mm diameter). After 1 min, 35 μL freshly prepared functional CsgA protein solution of different concentration (measured by BCA assay) was added onto mica and incubated for 24 h. Then the solution was removed by sucking up all the liquid that comes off in a single thumb-up movement while keeping the pipette attached to and almost perpendicular to the mica surface. After that, 100 μL of a 1×HEPES-Mg^{2+} was added onto the mica and the sample was imaged with AFM[3].

Assessing Replacement Reaction between CsgB and CsgA

A flow rate was applied at 25 μL/min to assess the interaction of the protein immobilized on the sensor surface with its surrounding proteins in solution, Specifically, the Ni-NTA-modified gold chip was initially incubated with CsgB-His for 3 hours. After washing away the loosely bound CsgB-His on the surfaces with copious amount of buffer, we applied GFP-His solution in the system to carry out the replacement reaction by incubating the CsgB-His tethered Ni-NTA modified gold chip in the presence of 3.0 μM GFP-His solution. We then reapplied large amount of buffer (with constant flow rate (100 μL/min) to wash away any loosely bound GFP-His protein.

For fluorescence imaging, the Ni-NTA modified gold surface was first incubated with CsgB-His in a region-defined manner, in which only half of the gold surfaces was exposed to excessive amount of CsgB-His proteins. We then applied excessive amount of 3.0 μM GFP-His solution to cover both areas and the substrate with covered solution was incubated at ambient condition for another 6 hours. The sample was then imaged with fluorescent microscopy.

SDS-PAGE (SDS-polyacrylamide gel electrophoresis)

Samples of washing solutions and eluents collected during the purification process were used for SDS/PAGE. Specifically, samples were mixed with loading sample buffer (NuPAGE, LDS sample buffer (4×)) in 1.5 mL microtubes and boiled for 90°C for 10 min. Afterwards, the samples, along with a protein ladder (NOVEX Sharp Pre-stained Protein Standard), were loaded into NuPAGE® 4-12% Bis-Tris gels. The samples were run at 120 volts for 45 mins using Nupage MES SDS running buffer (NOVEX) following standard SDS/PAGE procedures. The gels were then stained with Coomassie Blue for 30 min and were immersed in 25 mL destaining solutions (10% acetic acid / 40% Methanol / 50% DI water) for 30 min for three times. Afterwards, the gels were incubated in copious amounts of deionized water overnight and finally imaged using a Bio-Rad ChemiDoc MP system.

Western blotting

Samples were electrophoresed on 12% SDS-polyacrylamide gels and blotted onto polyvinylidene difluoride membranes using iBlot (Invitrogen). The protein ladder used in Western blotting was the same as for SDS/PAGE. Western blots were probed by primary anti-His mouse monoclonal antibody (TransGen, HT501-02) at a dilution of 1:5,000. Secondary goat anti-mouse antibodies lgG conjugated to horseradish peroxidase (HRP) (TransGen, HS201-01) was used at a dilution of 1:5,000. The blots were developed using the Pierce SuperSignal detection system and imaged using a Bio-Rad ChemiDoc MP system.

Analysis of CsgA Deamidation with Mass Spectrometry

1 μL CsgA stock solution was diluted into 50 μL solution containing 2 M urea and 0.1% trifluoroacetic acid, and passed through a custom-packed pepsin column for 1 min. The peptic peptides were trapped on the C8 column and then eluted with a linear gradient of 5−60% acetonitrile (ACN) with 0.1% FA at 50 μL/min for 5 mins.

Mass spectra were collected on a Thermo Q Exactive plus Orbitrap mass spectrometer (San Jose, CA) operated in a data-dependent acquisition mode (to sample the top 15 most abundant ions). Product-ion spectra were submitted to Byonic for identification (Protein Metrics, San Carlos, CA). The search results along with raw files were loaded into Byologic (Protein Metrics, San Carlos, CA) for manual validation and further analysis with their deamidation quantification feature enabled. The deamidation extent was calculated by dividing the areas of peaks (all charge states) representing a deamidation by the sum of all peak areas for the peptide. At each time point, two independent digestions and two injections into the mass spectrometer per sample were employed.

Thioflavin T (ThT) assay

Purified proteins (5.0 μM) were loaded on 96-well black plates with transparent bottoms. Dependent on the specific experiments, the purified protein solution was added either with or without CB-origami (10 nM). ThT was added with a final concentration of 20 μM. Fluorescence was measured every 3 min after shaking 5 sec by a BioTek Synergy H1 Microplate Reader using BioTek GEN5 software set to 438 nm excitation and 495 nm emission with a 475-nm cutoff at 25 ^{o}C.[4]

Fourier-transform Infrared Spectroscopy

FTIR was conducted using a Tensor 27 (Bruker) FTIR spectrophotometer. Approximately 4 μg of

protein was dried on the ATR crystal with dry nitrogen. Spectra were recorded from 4000 to 1000 cm^{-1} using a nominal resolution of 2 cm^{-1} and 64 accumulations. Identification of the amide I region of different samples was performed by second derivative analysis.

Atomic force microscopy imaging

Samples were first deposited on mica surfaces. AFM images were then taken in tapping mode either in fluid mode or air mode on a MFP-3D AFM (Asylum Research) using TR400PSA tips (Olympus). Typical scanning parameters were: scan rate =1-2 Hz, lines = 512, amplitude set point = 150-300 mV, drive amplitude =180–300 mV, integral gain = 18. High-speed atomic force microscopy (HS-AFM) imaging was carried out with Cypher VRS (Asylum Research) using AC10DS tips (Olympus). Typical scanning parameters were: scan rate = 30 Hz, lines = 256, amplitude set point = 150-300 mV, drive amplitude = 180-300 mV, integral gain = 18.

Image processing of CsgA fibrillation monitored with AFM

A kymograph construction starteding from a recorded video timelapse of in situ CsgA fibrillation. The image stack was resliced along a segmented line selection (y_0 to y_{end}) that follows the growth trajectory of the fibril as determined from the last image. At each time point (i), the pixel values along the fiber trajectory were linearized into a column. Columns were then stacked together to construct a kymograph that represented pixel evolution in space and time.

Transmission electron microscopy (TEM) imaging

TEM samples were prepared by dropping 2 μL of the purified sample solution on a carbon-coated grid (400 mesh, Ted Pella) that was previously negatively glow discharged using an Emitech K100X instrument. After 1 minute, the sample drop was wicked away from the grid with a piece of filter paper. The grid was then washed by a drop of water to remove the excess salt, and the excess water was again wicked away by a piece of filter paper. For negative staining, the grid was treated with a drop of 0.7 % uranyl formate solution for 2 seconds and excess solution was wicked away with a piece of filter paper. The grid was then treated with another drop of uranyl formate solution for 12 seconds, and the excess solution was removed by filter paper. The grid was kept at room temperature to allow drying for over 2 hours. TEM imaging was conducted using FEI Tecnai Spirit transmission electron microscope, operated at 120 kV in bright field mode.

Kinetic analysis with global fitting

Polymerization of functional amyloid proteins typically follows two assembly pathways, primary and secondary pathway. During polymerization, the integrated rate law describing the generation of total fibril mass, M(t), over time as a function of the initial conditions and the rate constants of the system is given by:

$$\frac{M(t)}{M(\infty)} = 1 - \left(\frac{B_+ + C_+}{B_+ + C_+ e^{\kappa t}} * \frac{B_- + C_+ e^{\kappa t}}{B_- + C_+ e^{\kappa t}}\right)^{\frac{k_\infty^2}{\kappa k_{-\infty}}} * e^{-k_\infty t} \qquad (1)$$

$M(\infty)$ is the final total fibril mass, t is the time, λ and κ is combined parameter that controls proliferation through primary and secondary pathways, respectively.

Supplementary equation (1) depends on the rate constants through two parameters, λ and κ, alone because

$$B_{\pm} = (k_{\infty} \pm k_{-\infty})/(2\kappa) \quad (2)$$

$$C_{\pm} = \pm\lambda^2/(2\kappa^2) \quad (3)$$

$$k_{\infty} = \sqrt{2\kappa^2/[n_2(n_2+1)]+2\lambda^2/n_c} \quad (4)$$

$$k_{-\infty} = \sqrt{2k_{\infty}^2 - 4C_{+}C_{-}\kappa\text{^}2} \quad (5)$$

Although several parameters, including microscopic rate constants for primary nucleation (*Kn*), elongation (K_+), depolymerization (K_{off}), fragmentation (K_-), and fibril-catalyzed secondary nucleation (K_2), are required to fully describe the complete assembly process, two key combinations of the rate constants, λ and κ, can be used to define much of the macroscopic behavior.

In particular, λ, related to the rate of formation of new aggregates through primary pathways (referred to as primary proliferation rate) is defined as:

$$\lambda = \sqrt{2K_n * K_+ * m_0{}^{nc}} \quad (6)$$

κ, related to the rate of formation of new aggregates through secondary pathways, (referred to as secondary proliferation rate) is defined as:

$$\kappa = \sqrt{2K_2 * K_+ * m_0{}^{n_2+1}} \quad (7)$$

The initial concentration of soluble monomers is m_0, and the reaction orders describing the dependencies of the primary and secondary pathways on the monomer concentration are n_c and n_2.

Global Analysis of Experimental Kinetic Data

The global fit for the two parameters, λ and κ (shown in Figure 2e), was performed using the analytical rate law Supplementary equation (1). Because the predominant mechanism for CsgA polymerization follows secondary pathways (in which the kinetic rate for secondary pathway is significantly higher than that for Primary pathway), in particular, via a monomer concentration-dependent secondary nucleation process, the value of n_2 and n_c in Supplementary Equation (6) and (7) should be 2[5, 6]. All global analytical fits were carried out using a Levenberg–Marquardt algorithm.

Supplementary Notes

Supplementary Note 1: The CsgA samples here refer to the His-Tag fused CsgA proteins, which had apparently normal capacity for self-assembly to form amyloid nanofibrils with typical cross-β structures and resembled the fibril structures present in wild type biofilms

Supplementary Note 2: A higher concentration, (for example, 5 μM), the polymerization already passed the initial nucleation stage and more independent fibrils formed and covered the surface of the substrate so that at higher fibril concentrations the DNA origami beneath the covered fibrils can't be resolved by AFM imaging.

Supplementary Note 3: We calculated the total number of all aggregates at various concentrations in the presence of CB-origami, (Total aggregate number = t * $K_{aggreates}$ = t * ($K_{primary}$ + $K_{secondary}$) =t ([CsgA] K_n + [CsgA] [Fiber] K_2). The model suggests that the number of CsgA aggregates based on independent nucleation events is significantly higher than that of CB-origami when the concentration of CsgA is over 3.0 μM (and the increase in the number of independent aggregates is not proportional to the increase of CsgA concentration), thus explaining the trend that a significantly increased number of self-assembled fibrils were indeed found on the mica surface under AFM imaging as CsgA concentration increased.

Supplementary Note 4: The kinetic model was constructed by applying different concentration of CsgA into the kinetic equation, with the kinetic parameters acquired from data based on ThT assay. Notably, this kinetic model is also consistent with the AFM results in Supplementary Figure S11.

Supplementary Note 5: These results clearly indicated that 3.0 μM GFP-His could not replace CsgB-His that was initially bound with Ni-NTA decorated Au surface. In addition, the CsgB-incubated area exhibited no GFP fluorescence feature while the other area was fully covered with GFP fluorescence, indicating that the GFP-His did not replace CsgB-His from the Ni-NTA decorated surfaces. However, our QCM-D experiments presented here were not ideal given the possibility that CsgB-his proteins tend to form nanofibers on the Au chip (even though the incubation time is short) and in light of the multivalent display of Ni- NTA sites on the chips. It is indeed very challenging to demonstrate if such a replacement equilibrium reaction does dominate or not in the system, particularly considering that the similarities in the molecular weight and structure between CsgB and CsgA.

Supplementary Note 6: To ensure clean samples for AFM imaging, we washed the AFM samples with water for 3 times to remove the impurities and salts on the mica surface. Afterwards, we applied tapping mode in air to record all the AFM images above. Nanofiber shrinkage arising from a dehydration effect with N_2 blowing during sample handling might occur. In addition, sample dehydration during tapping scanning mode in air as well as AFM tip artifacts (for example, the actual AFM tip radius may vary from batch to batch) would result in the morphological differences of fibril threads.

Supplementary References

1.Zhong, C. et al. Strong underwater adhesives made by self-assembling multi-protein nanofibres. *Nat. Nano.* **9**, 858-866 (2014).

2.Rothemund, P.W.K. Folding DNA to create nanoscale shapes and patterns. *Nature* **440**, 297-302 (2006).

3.Tikhomirov, G., Petersen, P. & Qian, L.L. Programmable disorder in random DNA tilings. *Nat. Nano.* **12**, 251-259 (2017).

4.Wang, X., Smith, D.R., Jones, J.W. & Chapman, M.R. In vitro polymerization of a functional Escherichia coli amyloid protein. *J. Biol. Chem.* **282**, 3713-3719 (2007).

5.Knowles, T.P.J. et al. An Analytical Solution to the Kinetics of Breakable Filament Assembly. *Science* **326**, 1533-1537 (2009).

6.Cohen, S.I.A. et al. Proliferation of amyloid-beta 42 aggregates occurs through a secondary nucleation mechanism. *Proc. Natl Acad. Sci. USA* **110**, 9758-9763 (2013).
