# Supplementary material for: Directing curli polymerization with DNA origami nucleators
Source: Nat Commun. 2019 Mar 27;10:1395. doi: 10.1038/s41467-019-09369-6 (PMC6437208; doi:10.1038/s41467-019-09369-6)
Supplement: Supplementary file 3 — Description of Additional Supplementary Files [file 41467_2019_9369_MOESM3_ESM.pdf]

Description of Additional Supplementary Files

File Name: Supplementary Data 1
Description: DNA sequences for Triangle Origami

File Name: Supplementary Movie 1
Description: In situ imaging of CsgA fiber nucleation on mica using Cypher VRS (Oxford): [CsgA] = 500 nM in 1×TAE 12.5 mM Mg^{2+} pH 8.0, 6 s/frame, total time: 2 min, 400 nm × 400 nm, Tapping Mode.

File Name: Supplementary Movie 2
Description: Typical departure mode of fibril elongation in situ imaging of CsgA fiber nucleation and growth on mica using Cypher VRS (Oxford): [CsgA] = 2.0 µM in 1×TAE 12.5 mM Mg^{2+} pH 8.0, 6 s/frame, total time: 2.5 min, 350 nm × 350 nm, Tapping Mode.

File Name: Supplementary Movie 3
Description: Typical arrival mode of fibril elongation in situ imaging of CsgA fiber nucleation and growth on mica using Cypher VRS (Oxford): [CsgA] = 2.0 µM in 1xTAE 12.5 mM Mg^{2+} pH 8.0, 6 s/frame, total time: 7 min, 350 nm × 350 nm, Tapping Mode.

File Name: Supplementary Movie 4
Description: Typical arrival mode of fibril elongation in situ imaging of CsgA fiber nucleation and growth on mica using Cypher VRS (Oxford): [CsgA] = 2.0 µM in 1×TAE 12.5 mM Mg^{2+} pH 8.0, 6 s/frame, total time: 7 min, 300 nm × 300 nm, Tapping Mode.

File Name: Supplementary Movie 5
Description: Typical fibril elongation in situ imaging of CsgA fiber nucleation and growth on mica using Cypher VRS (Oxford): [CsgA] = 2.0 µM in 1×TAE 12.5 mM Mg^{2+} pH 8.0, 6 s/frame, total time: 13 min, 1000 nm × 1000 nm, Tapping Mode.
