# Supplementary material for: Directing curli polymerization with DNA origami nucleators
Source: Nat Commun. 2019 Mar 27;10:1395. doi: 10.1038/s41467-019-09369-6 (PMC6437208; doi:10.1038/s41467-019-09369-6)
Supplement: Supplementary file 4 — Supplementary Data 1 [file 41467_2019_9369_MOESM4_ESM.docx]

| Oligo | Sequence |
| --- | --- |
| capature DNA-polyT | SH-TTTTTTTTTTTTTTTTTTTTTTTTTTT |
| capature DNA-polyGGT | TTTGGTGGTGGTGGTGGTGGTGGTGGT-SH |
| B32-A | AATACTGCGGAATCGTAGGGGGTAATAGTAAAATGTTTAGACTAAAAAAAAAAAAAAAAAAAAAAAAAAA |
| B41-B | CGACCTGCGGTCAATCATAAGGGAACGGAACAACATTATTAAAAAAAAAAAAAAAAAAAAAAAAAAA |
| B65-C | AAAACCACCACCACCACCACCACCACCCCTGACGAGAAACACCAGAACGAGTAGGCTGCTCATTCAGTGA |
| A32-D | CAGAAGGAAACCGAGGTTTTTAAGAAAAGTAAGCAGATAGCCGAAAAAAAAAAAAAAAAAAAAAAAAAAA |
| C32-E | TCTTTGATTAGTAATAGTCTGTCCATCACGCAAATTAACCGTTAAAAAAAAAAAAAAAAAAAAAAAAAAA |
| A01 | CGGGGTTTCCTCAAGAGAAGGATTTTGAATTA |
| A02 | AGCGTCATGTCTCTGAATTTACCGACTACCTT |
| A03 | TTCATAATCCCCTTATTAGCGTTTTTCTTACC |
| A04 | ATGGTTTATGTCACAATCAATAGATATTAAAC |
| A05 | TTTGATGATTAAGAGGCTGAGACTTGCTCAGTACCAGGCG |
| A06 | CCGGAACCCAGAATGGAAAGCGCAACATGGCT |
| A07 | AAAGACAACATTTTCGGTCATAGCCAAAATCA |
| A08 | GACGGGAGAATTAACTCGGAATAAGTTTATTTCCAGCGCC |
| A09 | GATAAGTGCCGTCGAGCTGAAACATGAAAGTATACAGGAG |
| A10 | TGTACTGGAAATCCTCATTAAAGCAGAGCCAC |
| A11 | CACCGGAAAGCGCGTTTTCATCGGAAGGGCGA |
| A12 | CATTCAACAAACGCAAAGACACCAGAACACCCTGAACAAA |
| A13 | TTTAACGGTTCGGAACCTATTATTAGGGTTGATATAAGTA |
| A14 | CTCAGAGCATATTCACAAACAAATTAATAAGT |
| A15 | GGAGGGAATTTAGCGTCAGACTGTCCGCCTCC |
| A16 | GTCAGAGGGTAATTGATGGCAACATATAAAAGCGATTGAG |
| A17 | TAGCCCGGAATAGGTGAATGCCCCCTGCCTATGGTCAGTG |
| A18 | CCTTGAGTCAGACGATTGGCCTTGCGCCACCC |
| A19 | TCAGAACCCAGAATCAAGTTTGCCGGTAAATA |
| A20 | TTGACGGAAATACATACATAAAGGGCGCTAATATCAGAGA |
| A21 | CAGAGCCAGGAGGTTGAGGCAGGTAACAGTGCCCG |
| A22 | ATTAAAGGCCGTAATCAGTAGCGAGCCACCCT |
| A23 | GATAACCCACAAGAATGTTAGCAAACGTAGAAAATTATTC |
| A24 | GCCGCCAGCATTGACACCACCCTC |
| A25 | AGAGCCGCACCATCGATAGCAGCATGAATTAT |
| A26 | CACCGTCACCTTATTACGCAGTATTGAGTTAAGCCCAATA |
| A27 | AGCCATTTAAACGTCACCAATGAACACCAGAACCA |
| A28 | ATAAGAGCAAGAAACATGGCATGATTAAGACTCCGACTTG |
| A29 | CCATTAGCAAGGCCGGGGGAATTA |
| A30 | GAGCCAGCGAATACCCAAAAGAACATGAAATAGCAATAGC |
| A31 | TATCTTACCGAAGCCCAAACGCAATAATAACGAAAATCACCAG |
| A32 | CAGAAGGAAACCGAGGTTTTTAAGAAAAGTAAGCAGATAGCCG |
| A33 | CCTTTTTTCATTTAACAATTTCATAGGATTAG |
| A34 | TTTAACCTATCATAGGTCTGAGAGTTCCAGTA |
| A35 | AGTATAAAATATGCGTTATACAAAGCCATCTT |
| A36 | CAAGTACCTCATTCCAAGAACGGGAAATTCAT |
| A37 | AGAGAATAACATAAAAACAGGGAAGCGCATTA |
| A38 | AAAACAAAATTAATTAAATGGAAACAGTACATTAGTGAAT |
| A39 | TTATCAAACCGGCTTAGGTTGGGTAAGCCTGT |
| A40 | TTAGTATCGCCAACGCTCAACAGTCGGCTGTC |
| A41 | TTTCCTTAGCACTCATCGAGAACAATAGCAGCCTTTACAG |
| A42 | AGAGTCAAAAATCAATATATGTGATGAAACAAACATCAAG |
| A43 | ACTAGAAATATATAACTATATGTACGCTGAGA |
| A44 | TCAATAATAGGGCTTAATTGAGAATCATAATT |
| A45 | AACGTCAAAAATGAAAAGCAAGCCGTTTTTATGAAACCAA |
| A46 | GAGCAAAAGAAGATGAGTGAATAACCTTGCTTATAGCTTA |
| A47 | GATTAAGAAATGCTGATGCAAATCAGAATAAA |
| A48 | CACCGGAATCGCCATATTTAACAAAATTTACG |
| A49 | AGCATGTATTTCATCGTAGGAATCAAACGATTTTTTGTTT |
| A50 | ACATAGCGCTGTAAATCGTCGCTATTCATTTCAATTACCT |
| A51 | GTTAAATACAATCGCAAGACAAAGCCTTGAAA |
| A52 | CCCATCCTCGCCAACATGTAATTTAATAAGGC |
| A53 | TCCCAATCCAAATAAGATTACCGCGCCCAATAAATAATAT |
| A54 | TCCCTTAGAATAACGCGAGAAAACTTTTACCGACC |
| A55 | GTGTGATAAGGCAGAGGCATTTTCAGTCCTGA |
| A56 | ACAAGAAAGCAAGCAAATCAGATAACAGCCATATTATTTA |
| A57 | GTTTGAAATTCAAATATATTTTAG |
| A58 | AATAGATAGAGCCAGTAATAAGAGATTTAATG |
| A59 | GCCAGTTACAAAATAATAGAAGGCTTATCCGGTTATCAAC |
| A60 | TTCTGACCTAAAATATAAAGTACCGACTGCAGAAC |
| A61 | GCGCCTGTTATTCTAAGAACGCGATTCCAGAGCCTAATTT |
| A62 | TCAGCTAAAAAAGGTAAAGTAATT |
| A63 | ACGCTAACGAGCGTCTGGCGTTTTAGCGAACCCAACATGT |
| A64 | ACGACAATAAATCCCGACTTGCGGGAGATCCTGAATCTTACCA |
| A65 | TGCTATTTTGCACCCAGCTACAATTTTGTTTTGAAGCCTTAAA |
| B01 | TCATATGTGTAATCGTAAAACTAGTCATTTTC |
| B02 | GTGAGAAAATGTGTAGGTAAAGATACAACTTT |
| B03 | GGCATCAAATTTGGGGCGCGAGCTAGTTAAAG |
| B04 | TTCGAGCTAAGACTTCAAATATCGGGAACGAG |
| B05 | ACAGTCAAAGAGAATCGATGAACGACCCCGGTTGATAATC |
| B06 | ATAGTAGTATGCAATGCCTGAGTAGGCCGGAG |
| B07 | AACCAGACGTTTAGCTATATTTTCTTCTACTA |
| B08 | GAATACCACATTCAACTTAAGAGGAAGCCCGATCAAAGCG |
| B09 | AGAAAAGCCCCAAAAAGAGTCTGGAGCAAACAATCACCAT |
| B10 | CAATATGACCCTCATATATTTTAAAGCATTAA |
| B11 | CATCCAATAAATGGTCAATAACCTCGGAAGCA |
| B12 | AACTCCAAGATTGCATCAAAAAGATAATGCAGATACATAA |
| B13 | CGTTCTAGTCAGGTCATTGCCTGACAGGAAGATTGTATAA |
| B14 | CAGGCAAGATAAAAATTTTTAGAATATTCAAC |
| B15 | GATTAGAGATTAGATACATTTCGCAAATCATA |
| B16 | CGCCAAAAGGAATTACAGTCAGAAGCAAAGCGCAGGTCAG |
| B17 | GCAAATATTTAAATTGAGATCTACAAAGGCTACTGATAAA |
| B18 | TTAATGCCTTATTTCAACGCAAGGGCAAAGAA |
| B19 | TTAGCAAATAGATTTAGTTTGACCAGTACCTT |
| B20 | TAATTGCTTTACCCTGACTATTATGAGGCATAGTAAGAGC |
| B21 | ATAAAGCCTTTGCGGGAGAAGCCTGGAGAGGGTAG |
| B22 | TAAGAGGTCAATTCTGCGAACGAGATTAAGCA |
| B23 | AACACTATCATAACCCATCAAAAATCAGGTCTCCTTTTGA |
| B24 | ATGACCCTGTAATACTTCAGAGCA |
| B25 | TAAAGCTATATAACAGTTGATTCCCATTTTTG |
| B26 | CGGATGGCACGAGAATGACCATAATCGTTTACCAGACGAC |
| B27 | TAATTGCTTGGAAGTTTCATTCCAAATCGGTTGTA |
| B28 | GATAAAAACCAAAATATTAAACAGTTCAGAAATTAGAGCT |
| B29 | ACTAAAGTACGGTGTCGAATATAA |
| B30 | TGCTGTAGATCCCCCTCAAATGCTGCGAGAGGCTTTTGCA |
| B31 | AAAGAAGTTTTGCCAGCATAAATATTCATTGACTCAACATGTT |
| B32 | AATACTGCGGAATCGTAGGGGGTAATAGTAAAATGTTTAGACT |
| B33 | AGGGATAGCTCAGAGCCACCACCCCATGTCAA |
| B34 | CAACAGTTTATGGGATTTTGCTAATCAAAAGG |
| B35 | GCCGCTTTGCTGAGGCTTGCAGGGGAAAAGGT |
| B36 | GCGCAGACTCCATGTTACTTAGCCCGTTTTAA |
| B37 | ACAGGTAGAAAGATTCATCAGTTGAGATTTAG |
| B38 | CCTCAGAACCGCCACCCAAGCCCAATAGGAACGTAAATGA |
| B39 | ATTTTCTGTCAGCGGAGTGAGAATACCGATAT |
| B40 | ATTCGGTCTGCGGGATCGTCACCCGAAATCCG |
| B41 | CGACCTGCGGTCAATCATAAGGGAACGGAACAACATTATT |
| B42 | AGACGTTACCATGTACCGTAACACCCCTCAGAACCGCCAC |
| B43 | CACGCATAAGAAAGGAACAACTAAGTCTTTCC |
| B44 | ATTGTGTCTCAGCAGCGAAAGACACCATCGCC |
| B45 | TTAATAAAACGAACTAACCGAACTGACCAACTCCTGATAA |
| B46 | AGGTTTAGTACCGCCATGAGTTTCGTCACCAGGATCTAAA |
| B47 | GTTTTGTCAGGAATTGCGAATAATCCGACAAT |
| B48 | GACAACAAGCATCGGAACGAGGGTGAGATTTG |
| B49 | TATCATCGTTGAAAGAGGACAGATGGAAGAAAAATCTACG |
| B50 | AGCGTAACTACAAACTACAACGCCTATCACCGTACTCAGG |
| B51 | TAGTTGCGAATTTTTTCACGTTGATCATAGTT |
| B52 | GTACAACGAGCAACGGCTACAGAGGATACCGA |
| B53 | ACCAGTCAGGACGTTGGAACGGTGTACAGACCGAAACAAA |
| B54 | ACAGACAGCCCAAATCTCCAAAAAAAAATTTCTTA |
| B55 | AACAGCTTGCTTTGAGGACTAAAGCGATTATA |
| B56 | CCAAGCGCAGGCGCATAGGCTGGCAGAACTGGCTCATTAT |
| B57 | CGAGGTGAGGCTCCAAAAGGAGCC |
| B58 | ACCCCCAGACTTTTTCATGAGGAACTTGCTTT |
| B59 | ACCTTATGCGATTTTATGACCTTCATCAAGAGCATCTTTG |
| B60 | CGGTTTATCAGGTTTCCATTAAACGGGAATACACT |
| B61 | AAAACACTTAATCTTGACAAGAACTTAATCATTGTGAATT |
| B62 | GGCAAAAGTAAAATACGTAATGCC |
| B63 | TGGTTTAATTTCAACTCGGATATTCATTACCCACGAAAGA |
| B64 | ACCAACCTAAAAAATCAACGTAACAAATAAATTGGGCTTGAGA |
| B65 | CCTGACGAGAAACACCAGAACGAGTAGGCTGCTCATTCAGTGA |
| Link-A1C | TTAATTAATTTTTTACCATATCAAA |
| Link-A2C | TTAATTTCATCTTAGACTTTACAA |
| Link-A3C | CTGTCCAGACGTATACCGAACGA |
| Link-A4C | TCAAGATTAGTGTAGCAATACT |
| Link-B1A | TGTAGCATTCCTTTTATAAACAGTT |
| Link-B2A | TTTAATTGTATTTCCACCAGAGCC |
| Link-B3A | ACTACGAAGGCTTAGCACCATTA |
| Link-B4A | ATAAGGCTTGCAACAAAGTTAC |
| Link-C1B | GTGGGAACAAATTTCTATTTTTGAG |
| Link-C2B | CGGTGCGGGCCTTCCAAAAACATT |
| Link-C3B | ATGAGTGAGCTTTTAAATATGCA |
| Link-C4B | ACTATTAAAGAGGATAGCGTCC |
| Loop | GCGCTTAATGCGCCGCTACAGGGC |
| C01 | TCGGGAGATATACAGTAACAGTACAAATAATT |
| C02 | CCTGATTAAAGGAGCGGAATTATCTCGGCCTC |
| C03 | GCAAATCACCTCAATCAATATCTGCAGGTCGA |
| C04 | CGACCAGTACATTGGCAGATTCACCTGATTGC |
| C05 | TGGCAATTTTTAACGTCAGATGAAAACAATAACGGATTCG |
| C06 | AAGGAATTACAAAGAAACCACCAGTCAGATGA |
| C07 | GGACATTCACCTCAAATATCAAACACAGTTGA |
| C08 | TTGACGAGCACGTATACTGAAATGGATTATTTAATAAAAG |
| C09 | CCTGATTGCTTTGAATTGCGTAGATTTTCAGGCATCAATA |
| C10 | TAATCCTGATTATCATTTTGCGGAGAGGAAGG |
| C11 | TTATCTAAAGCATCACCTTGCTGATGGCCAAC |
| C12 | AGAGATAGTTTGACGCTCAATCGTACGTGCTTTCCTCGTT |
| C13 | GATTATACACAGAAATAAAGAAATACCAAGTTACAAAATC |
| C14 | TAGGAGCATAAAAGTTTGAGTAACATTGTTTG |
| C15 | TGACCTGACAAATGAAAAATCTAAAATATCTT |
| C16 | AGAATCAGAGCGGGAGATGGAAATACCTACATAACCCTTC |
| C17 | GCGCAGAGGCGAATTAATTATTTGCACGTAAATTCTGAAT |
| C18 | AATGGAAGCGAACGTTATTAATTTCTAACAAC |
| C19 | TAATAGATCGCTGAGAGCCAGCAGAAGCGTAA |
| C20 | GAATACGTAACAGGAAAAACGCTCCTAAACAGGAGGCCGA |
| C21 | TCAATAGATATTAAATCCTTTGCCGGTTAGAACCT |
| C22 | CAATATTTGCCTGCAACAGTGCCATAGAGCCG |
| C23 | TTAAAGGGATTTTAGATACCGCCAGCCATTGCGGCACAGA |
| C24 | ACAATTCGACAACTCGTAATACAT |
| C25 | TTGAGGATGGTCAGTATTAACACCTTGAATGG |
| C26 | CTATTAGTATATCCAGAACAATATCAGGAACGGTACGCCA |
| C27 | CGCGAACTAAAACAGAGGTGAGGCTTAGAAGTATT |
| C28 | GAATCCTGAGAAGTGTATCGGCCTTGCTGGTACTTTAATG |
| C29 | ACCACCAGCAGAAGATGATAGCCC |
| C30 | TAAAACATTAGAAGAACTCAAACTTTTTATAATCAGTGAG |
| C31 | GCCACCGAGTAAAAGAACATCACTTGCCTGAGCGCCATTAAAA |
| C32 | TCTTTGATTAGTAATAGTCTGTCCATCACGCAAATTAACCGTT |
| C33 | CGCGTCTGATAGGAACGCCATCAACTTTTACA |
| C34 | AGGAAGATGGGGACGACGACAGTAATCATATT |
| C35 | CTCTAGAGCAAGCTTGCATGCCTGGTCAGTTG |
| C36 | CCTTCACCGTGAGACGGGCAACAGCAGTCACA |
| C37 | CGAGAAAGGAAGGGAAGCGTACTATGGTTGCT |
| C38 | GCTCATTTTTTAACCAGCCTTCCTGTAGCCAGGCATCTGC |
| C39 | CAGTTTGACGCACTCCAGCCAGCTAAACGACG |
| C40 | GCCAGTGCGATCCCCGGGTACCGAGTTTTTCT |
| C41 | TTTCACCAGCCTGGCCCTGAGAGAAAGCCGGCGAACGTGG |
| C42 | GTAACCGTCTTTCATCAACATTAAAATTTTTGTTAAATCA |
| C43 | ACGTTGTATTCCGGCACCGCTTCTGGCGCATC |
| C44 | CCAGGGTGGCTCGAATTCGTAATCCAGTCACG |
| C45 | TAGAGCTTGACGGGGAGTTGCAGCAAGCGGTCATTGGGCG |
| C46 | GTTAAAATTCGCATTAATGTGAGCGAGTAACACACGTTGG |
| C47 | TGTAGATGGGTGCCGGAAACCAGGAACGCCAG |
| C48 | GGTTTTCCATGGTCATAGCTGTTTGAGAGGCG |
| C49 | GTTTGCGTCACGCTGGTTTGCCCCAAGGGAGCCCCCGATT |
| C50 | GGATAGGTACCCGTCGGATTCTCCTAAACGTTAATATTTT |
| C51 | AGTTGGGTCAAAGCGCCATTCGCCCCGTAATG |
| C52 | CGCGCGGGCCTGTGTGAAATTGTTGGCGATTA |
| C53 | CTAAATCGGAACCCTAAGCAGGCGAAAATCCTTCGGCCAA |
| C54 | CGGCGGATTGAATTCAGGCTGCGCAACGGGGGATG |
| C55 | TGCTGCAAATCCGCTCACAATTCCCAGCTGCA |
| C56 | TTAATGAAGTTTGATGGTGGTTCCGAGGTGCCGTAAAGCA |
| C57 | TGGCGAAATGTTGGGAAGGGCGAT |
| C58 | TGTCGTGCACACAACATACGAGCCACGCCAGC |
| C59 | CAAGTTTTTTGGGGTCGAAATCGGCAAAATCCGGGAAACC |
| C60 | TCTTCGCTATTGGAAGCATAAAGTGTATGCCCGCT |
| C61 | TTCCAGTCCTTATAAATCAAAAGAGAACCATCACCCAAAT |
| C62 | GCGCTCACAAGCCTGGGGTGCCTA |
| C63 | CGATGGCCCACTACGTATAGCCCGAGATAGGGATTGCGTT |
| C64 | AACTCACATTATTGAGTGTTGTTCCAGAAACCGTCTATCAGGG |
| C65 | ACGTGGACTCCAACGTCAAAGGGCGAATTTGGAACAAGAGTCC |
